# Has the COVID-19 pandemic enhanced the capacity of rural public health emergency management? Evidence from Jiangsu Province of China

**DOI:** 10.3389/fpubh.2025.1670983

**Published:** 2026-01-12

**Authors:** Ming Cao, Yajing Zhang

**Affiliations:** School of Public Policy & Management, China University of Mining and Technology, Xuzhou, China

**Keywords:** COVID-19, emergency management capabilities, public health emergencies, rural public health, rural public health emergency management

## Abstract

Public health emergencies pose both challenges and opportunities for improving rural public health emergency management capacity. This study develops a comprehensive evaluation framework comprising 22 indicators across four dimensions: emergency infrastructure, emergency preparedness, emergency response, and emergency recovery. Using expert scoring, the analytic hierarchy process (AHP), and the entropy weight method, the rural public health emergency management capacity of Jiangsu Province was quantitatively assessed for the period 2016–2023 to compare changes before and after the COVID-19 pandemic. The results show that the outbreak of COVID-19 significantly enhanced the overall capacity of rural public health emergency management in Jiangsu Province, with the composite score increasing from 4.97 to 6.84. Among the four dimensions, emergency infrastructure capacity experienced the most pronounced improvement, rising from 4.02 to 9.66, whereas gains in emergency preparedness, response, and recovery capacities were relatively limited. These findings indicate that major public health emergencies can accelerate infrastructure development but do not automatically strengthen institutional preparedness or recovery mechanisms in rural areas. Accordingly, this study suggests increasing financial investment, promoting multi-actor collaborative governance, and improving public health emergency management mechanisms to further enhance rural public health emergency capacity in China.

## 1 Introduction

In the context of a risk society, public health emergencies are characterized by suddenness, mass, comprehensiveness and systematicness in handling. The COVID-19 pandemic at the end of 2019 brought a major test to humanity, seriously threatening human life and social stability, and the economic, social, cultural and political risks that evolved from it brought incalculable losses to humanity. The national governance system and governance capacity are not only reflected in regular governance activities, but also in the level of handling major public health crises (1). During this battle against the epidemic, various regions in China have successively organized and carried out emergency plans for public health emergencies, achieving a series of remarkable results in the fight against the epidemic. At the same time, this has fully exposed the serious shortcomings of emergency management and capacity building for public health emergencies in rural areas of China at present.

Rural public health emergency response capacity is the comprehensive ability demonstrated by relevant governments or organizations in rural areas when dealing with public health emergencies. It is the comprehensive handling ability to utilize, coordinate and integrate all resources from the beginning of emergency preparedness through detection and early warning, in order to achieve effective response in emergency response and post-disaster recovery. China is a major agricultural country. As of 2021, the rural population in China was as high as 498.35 million, accounting for 35% of the total population. Rural public health has always been the focus of China’s public health efforts. The establishment and improvement of rural public health emergency management is one of the core essentials of building a modern rural governance system, which includes two dimensions: the state’s general governance of rural areas and the internal governance of rural areas. The spread of COVID-19 in 2019 exposed problems such as backward medical facilities in rural areas, shortage of emergency reserve materials, weak awareness of health and epidemic prevention among villagers, and insufficient decision-making and leadership capabilities of grassroots organizations. Instead of having a positive effect, it provided more convenient channels for the spread of the epidemic. It sounded the alarm on rural public health emergency management capabilities (2–4), highlighting the huge gap between the rural emergency management system and the demands of the risk society. The lack of medical resources and the insufficiency of emergency response capabilities in rural areas have left them lagging behind urban areas in responding to public health emergencies. Therefore, public policy makers and public administration scholars in various countries need to focus on enhancing the resilience of public health emergency systems and the efficiency of emergency responses in rural areas. This paper takes Jiangsu Province as an example to analyze and explore the changing trends and internal logic of rural public health emergency management capabilities from 2016 to 2023 by constructing a multi-dimensional and whole-process emergency management capacity assessment framework and combining AHP and entropy weight methods. The aim is to provide practical references for improving the rural public health emergency response system and enhancing the governance level of emergencies.

## 2 Literature review

The key to dealing with major public health emergencies is to build emergency management capabilities in response to public health emergencies. The outbreak of COVID-19 has drawn widespread attention, and the academic community has conducted in-depth research on how to enhance public health emergency management capabilities. It mainly focuses on three aspects: research on the construction of rural public health systems, research on the emergency management capacity of rural public health events, and the impact of the COVID-19 pandemic on emergency management in rural areas.

### 2.1 Research on the construction of rural public health systems

Lyore et al. quantitatively studied 47 emergency plans in eastern North Carolina, USA, and found that these plans did not reflect the special needs of migrant workers, indicating a gap between emergency planning and the actual situation in rural areas and the need to further improve the adaptability of emergency plans (5). Sosin and Carpenter-Song cited rural New England as an example, where the government has long underfunded rural public health and neglected the construction of rural public health, resulting in a higher number of deaths during the pandemic than in cities (6). Leider et al. compared data from rural and urban areas in the United States over 40 years and found that the government tended to focus on the health status of the urban population and increase urban public health spending, but paid less attention to rural areas, resulting in a double gap between urban and rural areas, which led to the widening gap in life expectancy per capita between urban and rural areas (7). Hassan and other scholars, after conducting field research on the operation of federally Qualified health centers (FQHCs), pointed out that primary care institutions have practical difficulties such as weak emergency response capabilities and inadequate staff training (8). Li and Mostafav were able to accurately identify areas that needed focused emergency response by analyzing data on citizens’ behavior trajectories before the risk occurred, providing data support for the allocation of emergency resources in rural areas (9). Weber et al. found from a psychological perspective that individuals who have experienced disaster risk attach more importance to preparedness for prevention, indicating that the popularization of emergency knowledge and the conduct of emergency drills can enhance the disaster prevention awareness of rural residents and improve their resilience (10). Our findings echo this point, as the preparedness capacity in Jiangsu improved steadily during 2016–2023, though at a slower pace than infrastructure, suggesting that awareness and training require longer-term institutional support.

### 2.2 Research on emergency management capacity for sudden health incidents in rural areas

Sandra et al. determining risk indices across regions using the COVID-19 Community Vulnerability Index (CCVI) as a tool (11). European Union member states actively engage in disaster management by providing various disaster or appropriate education programs (EP) and participating in global activities, drawing lessons from COVID-19 and applying out-of-the-box critical thinking, and global leaders need to shift from isolated decision-making approaches to accepting multidisciplinary and cross-disciplinary collaboration. Koo et al. tuned the local influenza epidemic simulation model to apply theory to practice and estimated the likelihood of human-to-human transmission of severe acute respiratory syndrome coronavirus 2 (SARS-CoV-2) in the simulated Singaporean population (12). Kandel et al. used data and information from the Annual reporting tool for States Parties to the International Health Regulations to review existing levels of health security capabilities to prevent, detect, respond and build an enabling function for an effective response, as well as preparedness for action in response to public health risks and events (13). Yoon et al. used the World Health Organization’s International Health Regulations (IHR) monitoring tool and employed descriptive statistics and SWOT analysis to systematically assess the outcomes of practices in the implementation of the health regulations in various countries (14). This study provides a clear understanding of the strengths, weaknesses, opportunities and threats in rural areas when dealing with public health events, and also offers concrete support for rural areas to develop contingency plans. Xia et al. applied artificial intelligence technology to emergency management, using algorithms to schedule emergency resources and ensure the fairness of resource allocation. Therefore, it is necessary to increase the allocation of emergency resources in rural areas to deal with public health emergencies (15). Noelte et al. constructed a framework for public health emergency response preparedness, which includes planning, preparation, response, and recovery, providing a reference for establishing a scientific and systematic emergency management system in rural areas (16). This study extends such work by adopting a full-process, multi-dimensional evaluation system. The results show that while Jiangsu’s response and recovery capacities improved after COVID-19, they grew more slowly than infrastructure, highlighting the structural imbalances that previous literature has often theorized but rarely demonstrated with longitudinal evidence.

### 2.3 Impact of the COVID-19 pandemic on public health emergency management in rural areas

The outbreak of COVID-19 has severely impacted the rural public health emergency response system. Scholars have mainly studied the changes in rural public health emergency management before and after the COVID-19 pandemic from the perspectives of interdepartmental collaboration, resource allocation, technology empowerment, and organizational communication.

Rivera et al. analyzed the emergency management situation in Salvadoran during the pandemic and found that due to insufficient resource allocation at the grassroots level, there were problems such as poor information communication and unclear boundaries of responsibilities in cross-departmental cooperation, resulting in slow emergency response at the grassroots level (17). Alcendor et al. studied the situation in Tennessee after the end of the COVID-19 pandemic and found that vulnerable groups had difficulty continuing to receive medical services, proposing to protect the health rights and interests of vulnerable groups in the long term (18). Singh et al. found through a survey of the California Department of Public Health that members of the department were not adequately prepared in terms of training, organizational communication, etc., which seriously affected the effectiveness of emergency response. Training efforts should be intensified, especially for leaders (19). Bekemeier et al., taking rural areas in the northwestern United States as an example, revealed that rural areas had a significant adverse impact on emergency decision-making during the pandemic due to difficulties in obtaining public health data, imperfect data information sharing mechanisms, and weak data analysis capabilities (20). They suggested increasing resource input, improving data facility construction, and training grassroots digital personnel to improve the situation. Zhang et al. found that in the context of the COVID-19 pandemic, rural primary health workers in China were affected by professional identity, job satisfaction and occupational burnout, which threatened the stability of the rural primary health system (21). Stolerman et al. built a digital tracking model based on Internet technology to monitor the trend time of outbreaks in counties across the United States in real time, providing a new technical approach for epidemic monitoring (22). Biesiadecki, Hoff et al. suggested that the emergency response at the grassroots level should shift from passive response to active prevention, and proposed to improve the community participation mechanism and build resilient primary health institutions (20, 23).

At present, relevant scholars have conducted relatively rich research on the construction of rural public health emergency management. In terms of the construction of rural public health systems, problems such as insufficient personnel reserves, shortage of emergency resources and weak institutional guarantees in rural areas have been revealed, and it is suggested to enhance the capacity of rural emergency management by strengthening cross-departmental cooperation and using data technology, etc. In terms of emergency management capacity for public health emergencies in rural areas, scholars have improved the efficiency of rural emergency management by establishing an index evaluation system, using international monitoring tools, applying intelligent algorithms, etc., from the three aspects of governance system, technical path and institutional mechanism; In the study of the evolution of rural emergency management systems before and after the pandemic, an increasing number of scholars have begun to focus on the impact of the pandemic on rural governance models, resource allocation methods, and coordination mechanisms. Our evidence supports this shift: in Jiangsu, COVID-19 coincided with not only a surge in infrastructure investment but also the institutionalization of preparedness and response mechanisms, consistent with calls in the literature for more systematic and collaborative rural governance.

To sum up, existing research lacks a dynamic emergency management capacity assessment system based on the whole-process perspective and a systematic analysis of the changes in rural emergency management capacity before and after the epidemic with long-term sequential data. Compared with existing studies, the research contribution of this paper: First, a multi-dimensional assessment system for rural public health emergency management capacity has been constructed. Based on a review of the literature and in light of the current situation of rural public health emergency response in China, an evaluation index system for rural public health emergency management capacity, including emergency infrastructure capacity, emergency preparedness capacity, emergency response capacity and emergency recovery capacity, was developed to conduct a relatively comprehensive assessment of the emergency response capacity for rural public health emergencies; Secondly, a dynamic comparative analysis was carried out. This paper, with 2020 as the time frame, divided into two phases before and after the outbreak, conducts a horizontal and vertical comparison of rural public health emergency management capabilities, with the aim of revealing the impact of the COVID-19 pandemic on rural emergency systems. In particular, the structural differences we observed that rapid infrastructure growth alongside slower preparedness and recovery provide empirical confirmation of concerns raised in earlier studies about uneven capacity development in rural areas.

## 3 Methods and data

In this study, the evaluation index system for rural public health emergency management capacity was divided into four criterion layers: emergency infrastructure capacity, emergency preparedness capacity, emergency response capacity, and recovery capacity, and 22 index layers. Based on the relevant data of rural public health in Jiangsu Province over the past 8 years, following the principle of scientificity, the evaluation index system of rural public health emergency management capacity was analyzed using entropy weight method, analytic hierarchy process combined with expert scoring method. This combination of qualitative and quantitative methods has been widely applied in the research of emergency management capacity evaluation.

### 3.1 Construction of index system

**Figure 1:**
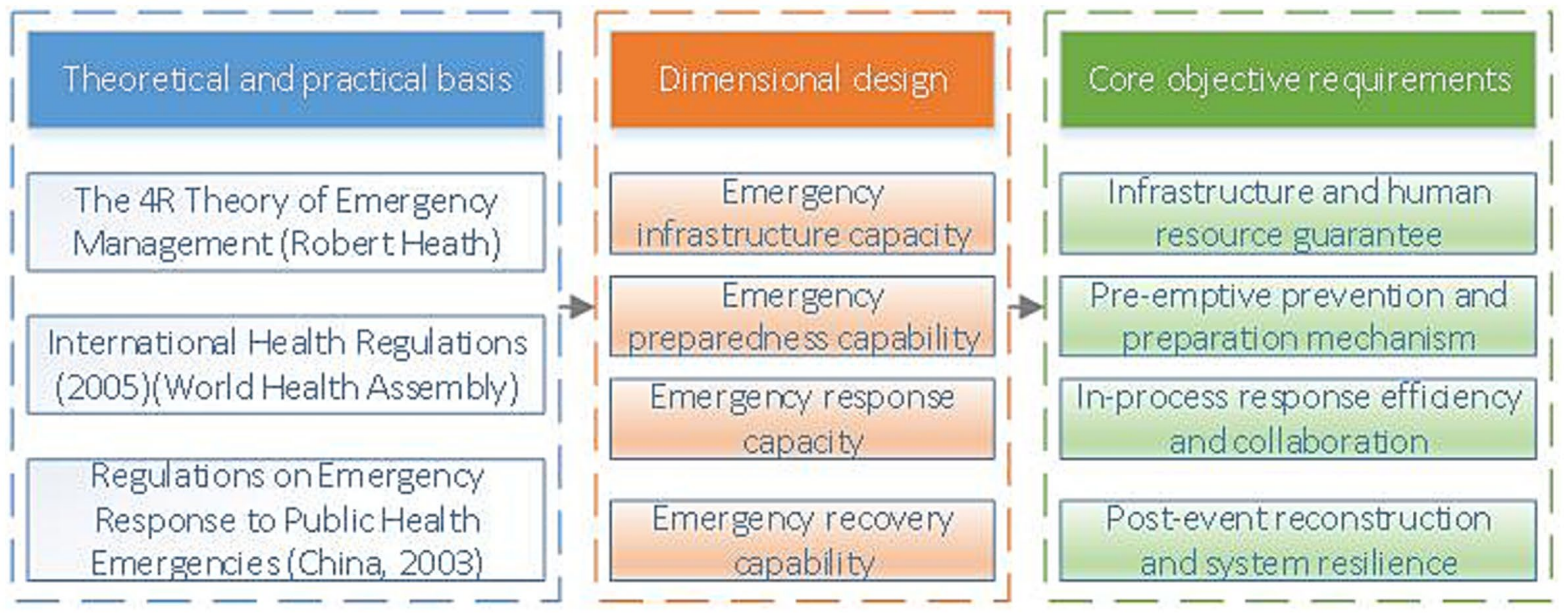
Design ideas for the evaluation dimensions of rural public health emergency management capabilities.

The improvement of emergency response capabilities is a complex and systematic project involving the coordinated efforts of multiple departments such as healthcare, publicity, and security, and encompasses multi-dimensional resource integration and scheduling. This study is based on Robert Heath ‘4R theory of crisis management, namely Reduction, Readiness, Response, and Recovery stages (24), combined with the capability model. Referring to relevant laws and regulations such as the International Health Regulations and the Regulations on Emergency Response to Public Health Emergencies, and in light of the actual situation in China, strictly following the principles of representativeness and scientificity of evaluation indicators, based on the publicly available data of rural areas in Jiangsu Province and expert scoring, the entropy weight method and analytic hierarchy process were used to construct the capacity system of rural public health emergency management, as shown in Figure 1. Starting from the four dimensions of resource, Preparedness, Response, and Recovery, and based on 22 basic indicators, supported by the data of Jiangsu rural areas from 2016 to 2023, Measure the current level of public health emergency management capacity (RPHE) in rural areas. This paper defines rural public health emergency management capacity as RPHE (shown in Table 1).

**Table 1:** Evaluation index system for rural public health emergency management capacity.

Target level	Criteria layer	Metrics layer	Relevance	Metric types
Rural Public Health Emergency Management Capacity (RPHE)	Emergency infrastructure capacity (Infr)	Infr1: Number of hospital beds per thousand population (beds)	Positive	Quantification
		Infr2: Township health centers per 10,000 people (number)	Positive	Quantification
		Infr3: Average number of household cars per 100 rural households (vehicles)	Positive	Quantification
		Infr4: Rural health technicians per thousand population (person)	Positive	Quantification
		Infr5: Number of physicians per 10,000 people (people)	Positive	Quantification
		Infr6: Per capita road area in County Town (square meters)	Positive	Quantification
	Emergency preparedness capacity (Prep)	Prep1: Community health service centers per 10,000 people (units)	Positive	Quantification
		Prep2: Proportion of household health care spending (%)	Negative	Quantification
		Prep3: The completeness of contingency plans	Positive	Qualitative
		Prep4: The effectiveness of public health advocacy	Positive	Qualitative
		Prep5: Completeness of emergency team members	Positive	Qualitative
		Prep6: Completeness of public health funding	Positive	Qualitative
	Emergency response capacity (Res)	Res1: Number of primary health care institutions (units)	Positive	Quantification
		Res2: Number of rural health check-ups (people)	Positive	Quantitative
		Res3: Government organization coordination leadership	Positive	Qualitative
		Res4: Number of mobile phones per 100 rural households (units)	Positive	Quantification
		Res5: Rural broadband access users (10,000 households)	Positive	Quantification
	Emergency recovery capacity (Rec)	Rec1: Minimum living allowance for rural residents (persons)	Negative	Quantification
		Rec2: Per capita consumption expenditure of rural residents (yuan)	Positive	Quantification
		Rec3: Number of people participating in basic health insurance (people)	Positive	Quantification
		Rec4: Rural per capita disposable income (yuan)	Positive	Quantification
		Rec5: Degree of recovery after a public health event	Positive	Qualitative

Reduction refers to the ability to enhance the allocation of basic resources and reduce the likelihood of public health risks occurring; Readiness encompasses the completeness of pre-emptive measures such as emergency response plans, public awareness campaigns, training, and funding; Response refers to the ability to rapidly respond, coordinate, communicate information, and allocate resources following a crisis; Recovery specifically pertains to the ability to ensure livelihood support, medical assistance, and the restoration of social order in the aftermath of a public health event. Among these, resource capacity serves as the material foundation of the entire emergency response system, reflecting the system’s supply capabilities in terms of preemptive prevention and routine safeguards, aligning with the “Reduction” component of the 4R theory; Preparedness capacity encompasses the development of emergency response plans, team building, funding allocation, and publicity and training, embodying the core requirements of “Readiness”; Response capacity focuses on response execution during emergencies, including organizational coordination, healthcare response, and information communication capabilities, corresponding to “Response”; Recovery capacity reflects the economic and health recovery capabilities of rural areas following emergencies, directly embodying the concept of “Resilience.”

### 3.2 Data sources

Based on the constructed evaluation index of rural public health emergency management capacity, the rural areas of Jiangsu Province from 2016 to 2023 were taken as the research objects, and the corresponding data were collected by consulting Jiangsu Statistical Yearbook, China Rural Statistical Yearbook, China Health Statistical Yearbook, etc. Some data (Prep3, Prep4, Prep5, Prep6, Res3, Rec5) are difficult to obtain directly from public data and require the assignment and scoring of 12 experts familiar with rural public health work. The scores obtained were standardized and incorporated into the model to enhance the scientificity of the indicator system. To further reduce the subjectivity of expert scoring, this study not only exercised strict control in expert selection and training, but also suggests that future research could adopt a triangulation approach—combining questionnaire surveys, field interviews, and secondary statistical data—to enhance the robustness and reliability of results.

### 3.3 Weight calculation

Frontiers requires figures to be submitted individually, in the same order as they are referred to in the manuscript. Figures will then be automatically embedded at the bottom of the submitted manuscript. Kindly ensure that each table and figure is mentioned in the text and in numerical order. Figures must be of sufficient resolution for publication. Figures which are not according to the guidelines will cause substantial delay during the production process. Figure legends should be placed at the end of the manuscript.

#### 3.3.1 Criterion layer weight calculation

The Analytic Hierarchy Process (AHP) is an operations research theory proposed by American operations researcher T. L. Saaty, which divides the various elements related to a problem into objective layer, criterion layer, scheme layer, etc., and conducts qualitative and quantitative analysis on this basis. It brings great convenience for relevant decision-makers to analyze problems that are difficult to quantify. The Analytic Hierarchy Process mainly consists of four steps: (a) establishing a hierarchical model; (b) Construct the judgment matrix; (c) Hierarchical single sorting and its consistency test; (d) Hierarchical total sorting and its consistency test.

There are many factors influencing rural public health emergency management capacity. The Analytic hierarchy process can be used to further categorize the factors influencing rural public health emergency management capacity into individual components, and then determine their relative importance through pairwise comparisons among the components. This study takes the evaluation index system of rural public health emergency management capacity as the target layer, and relevant experts assign weights to the four criterion layers of emergency infrastructure capacity, emergency preparedness capacity, emergency response capacity, and emergency recovery capacity and determine the judgment matrix. Construct the judgment matrix of the criteria layer of the rural public health emergency management capacity evaluation index system based on the hierarchical analysis comparison scale table.

In terms of expert selection, this study adheres to the principles of professionalism and representativeness. A total of 12 experts were invited to participate in the scoring work. These 12 experts come from Nanjing City, Huai’an City, Changzhou City, Xuzhou City and Taizhou City, respectively. All of them have more than 3 years of experience in rural grassroots public health work and are familiar with the public health emergency management process. In terms of the scoring process, the research team first elaborately explained the research purpose and the principle of the Analytic Hierarchy Process to the experts, provided a manual for interpreting the meanings of the indicators, and ensured the consistency of their understanding. Subsequently, expert scoring was carried out through questionnaires, pairwise comparisons of the criteria layer factors were made, and a judgment matrix was constructed based on scales 1 to 9. All scores were independently completed and submitted anonymously. The research team collected them uniformly and calculated the average value to minimize the impact of individual deviations. To reduce the subjective bias in expert scoring, the following control measures were adopted in this study: First, the multi-expert averaging method was used. By taking the average of the scores given by 12 experts, the stability of the results was improved; Second, conduct unified training and explanations before scoring to ensure that experts have a consistent understanding of the meanings of each indicator and the scoring rules. Thirdly, the validity of the judgment matrix is verified by using the consistency test method. If the consistency ratio CR is greater than 0.1, experts are required to re-evaluate to ensure that the judgment matrix has reasonable consistency. Ultimately, this study employs the root square method to calculate the eigenvectors of the criterion layer judgment matrix, obtaining the relative weights of each criterion, which are used to construct an evaluation model for rural public health emergency management capabilities.

Based on the constructed judgment matrix (Table 2), calculate its eigenvalues and eigenvectors using MATLAB, then normalize the eigenvectors to obtain the weights of the four criterion layers, and then conduct consistency tests according to the formula. From the test results, all the first-level indicators meet the requirements. The weights are obtained after scoring by 12 experts (Table 3).

**Table 2:** Scoring results for the target layer.

Scoring result	Infr	Prep	Res	Rec
Infr	1	1/1.5	1/2.5	1/14
Prep	1.5	1	1/2.1	1/1.1
Res	2.5	2.1	1	1/0.75
Rec	1.4	1.1	0.75	1

**Table 3:** Results of AHP hierarchy analysis.

Indicators	Feature vector	Weight values (%)	Maximum characteristic root	CI value
Infr	0.626	15.652	4.018	0.006
Prep	0.851	21.273		
Res	1.540	38.497		
Rec	0.983	24.577		

The weight calculation results of the Analytic Hierarchy Process show that the weight of emergency infrastructure capacity is 15.652%, that of emergency preparedness capacity is 21.273%, that of emergency response capacity is 38.497%, and that of emergency recovery capacity is 24.577%.

Due to the subjective and ambiguous nature of the judgments given by the 12 experts on various factors, and the accumulation of minor errors in the judgment comparison matrix leading to the contradiction of the judgment matrix, which in turn causes the unreliability of the judgment results, it is necessary to conduct consistency tests, that is, calculate the consistency index CR value (CR = CI/RI). The consistency index (CI) value is 0.006, the random consistency index (RI) is 0.89, and the consistency ratio (CR) value is 0.007 less than 0.1. The consistency of this judgment matrix is satisfactory, and the consistency test results pass (Table 4).

**Table 4:** Results of the consistency test.

Maximum eigenroot	CI value	RI value	CR value	Consistency test results
4.018	0.006	0.890	0.007	Passed

#### 3.3.2 Metric layer weight calculation

The entropy weight method is a technique that assigns weights to indicators based on the amount of information provided by the entropy values of each indicator (i.e., the degree of dispersion). The entropy of an indicator reflects its degree of dispersion in the overall research object, and its degree of dispersion is inversely proportional to its information entropy, and its weight is proportional to its degree of dispersion; If the values of an indicator are all equal, then the degree of dispersion of that indicator is zero and it does not work in the overall evaluation.

To eliminate dimensional differences and unify the evaluation scale, the Threshold Method was used for data standardization in this study. The threshold setting principle is: for positive indicators, the minimum value is 1 point and the maximum value is 10 points; For negative indicators, the minimum worth is 10 points and the maximum worth is 1 point. In the entropy method rule, the larger the positive indicator, the better; the smaller the negative indicator, the better. Among the 22 indicators selected in this study, there are both positive and reverse indicators, and the positive and reverse indicators are processed separately.

If it is a positive indicator, the processing formula is is in Equation (1):


(1) $$x_{\mathrm{ij}}^{\prime }=1+9\times \frac{x_{\mathrm{ij}}-\min(x_{j})}{\max(x_{j})-\min(x_{j})}$$

If it is a reverse indicator, the processing formula is in Equation (2):


(2) $$x_{\mathrm{ij}}^{\prime }=1+9\times \frac{\max(x_{j})-x_{\mathrm{ij}}}{\max(x_{j})-\min(x_{j})}$$

Note: *i* = 1, 2,… 8 represents the year (2016–2023); j∈{Infr1, Infr2,… Rec5} represents a set of 22 indicator codes; *x_ij_*: The original value of the *i*-th sample (year) on the *j*-th indicator; $x_{\mathrm{ij}}^{\prime }$ : Standardized score, range [1, 10].

The results are shown in Table 5.

**Table 5:** Results of standardized processing.

Year	2023	2022	2021	2020	2019	2018	2017	2016
Infr1	10.00	9.25	8.70	7.85	6.20	4.65	2.20	1.00
Infr2	7.50	7.50	10.00	10.00	10.00	7.50	5.50	1.00
Infr3	10.00	6.85	6.14	1.76	1.31	1.00	2.73	1.25
Infr4	10.00	9.27	8.85	8.23	6.50	4.54	2.00	1.00
Infr5	10.00	8.71	7.96	7.43	7.43	4.64	2.61	1.00
Infr6	10.00	6.31	5.81	4.72	3.73	3.05	2.97	1.00
Prep1	10.00	8.50	7.75	7.00	6.25	5.50	4.38	1.00
Prep2	4.00	6.57	8.29	1.00	3.57	4.86	6.14	10.00
Prep3	7.43	8.07	10.00	7.43	4.21	2.93	2.93	1.00
Prep4	8.65	9.26	10.00	6.83	4.65	3.26	1.74	1.00
Prep5	8.50	6.00	6.75	8.50	6.75	6.00	4.25	1.00
Prep6	8.00	6.25	9.00	7.50	4.50	4.50	1.00	1.00
Res1	10.00	6.69	5.88	4.95	3.74	1.00	1.00	1.00
Res2	6.18	6.21	10.00	4.36	4.64	4.04	2.21	1.00
Res3	8.00	8.00	10.00	9.00	6.00	4.00	2.50	1.00
Res4	10.00	6.70	7.20	6.30	4.30	4.10	2.80	1.00
Res5	9.80	8.00	10.00	7.43	6.55	4.35	2.71	1.00
Rec1	10.00	9.95	8.85	7.90	7.00	5.00	1.00	1.00
Rec2	10.00	7.67	6.30	2.43	3.09	2.00	1.11	1.00
Rec3	5.00	4.32	2.00	10.00	8.33	6.00	4.00	1.00
Rec4	10.00	8.44	7.12	5.12	3.93	2.51	1.20	1.00
Rec5	10.00	9.25	8.70	7.85	6.20	4.65	2.20	1.00

Based on the results of the standardization process, the entropy weight method was used to calculate the weights of the relative indicators in each dimension for the 22 indicator layers. The results are shown in Table 6.

**Table 6:** Final weights of the rural public health emergency management capacity evaluation system.

Criteria layer	Weights (W_ahp_)	Indicator layer	Weights (w_j_)
Infr	0.1565	Infr1	0.1463
		Infr2	0.1369
		Infr3	0.1726
		Infr4	0.1675
		Infr5	0.1631
		Infr6	0.2136
Prep	0.2127	Prep1	0.1618
		Prep2	0.1883
		Prep3	0.1592
		Prep4	0.1725
		Prep5	0.1527
		Prep6	0.1655
Res	0.3850	Res1	0.2079
		Res2	0.2015
		Res3	0.1993
		Res4	0.1936
		Res5	0.1977
Rec	0.2458	Rec1	0.2058
		Rec2	0.1983
		Rec3	0.2017
		Rec4	0.1962
		Rec5	0.1980

Calculate the feature gravity matrix in Equation (3):


(3) $$p_{\mathrm{ij}}=\frac{\mathrm{xij}\prime }{\sum_{i=1}^{m}\;\;x_{\mathrm{ij}}}$$

Calculate the entropy of the metric in Equation (4):


(4) $$e_{j}=-\frac{1}{\ln m}\;\sum_{i=1}^{m}\;\;p_{\mathrm{ij}}\;\ln p_{\mathrm{ij}}$$

Calculate the indicator difference coefficient in Equation (5):


(5) $$g_{j}=1-e_{j}$$

Calculate the metric weights in Equation (6):


(6) $$w_{j}=\frac{g_{j}}{\sum_{k=1}^{n}\;\;g_{k}}$$

Note: m = 8 (years of sample size); *n* = 22 metrics.

### 3.4 Comprehensive score calculation

The combined score is calculated based on the weights of the criterion layer and the indicator layer obtained above. The formulas are in Equations (7–9), and the results are shown in Table 7:


(7) $$W_{j}^{a}=W_{\mathrm{ahp}}\times w_{j}$$

(8) $$S_{\mathrm{zi}}=\sum_{j\in z}(\mathrm{xij}\prime \times w_{j})$$

(9) $$\;S_{i}=\sum_{j=1}^{n}\;\;(\;\mathrm{xij}\prime \times W_{j}^{a}\;)$$

**Table 7:** Comprehensive scores of the evaluation system for rural public health emergency management capacity.

Year	Infr	Prep	Res	Rec	RPHE
2023	9.66	7.23	7.21	7.05	6.84
2022	8.57	6.89	6.78	6.32	6.22
2021	8.24	7.58	6.99	6.73	6.53
2020	6.87	6.21	6.12	5.92	5.69
2019	6.64	6.05	6.32	6.02	5.77
2018	5.21	5.73	5.87	5.56	5.10
2017	4.11	5.42	5.35	5.18	4.88
2016	4.02	5.12	5.18	5.01	4.97

Note: z∈{Infr, Prep, Res, Rec}.

## 4 Results and discussion

### 4.1 Results analysis

Based on the results obtained, in the criteria layer of the evaluation index system for rural public health emergency management capacity, the final weight ranking is Res > Rec > Prep>Infr. Among them, the emergency infrastructure capacity index has the highest weighting, reaching 38.497%, and plays an important role in rural public health emergency management capacity. While the weight of the emergency infrastructure capacity indicator is relatively low at 24.577%, it reflects less of the rural public health emergency management capacity. In addition, the weights of emergency recovery capacity and emergency preparedness capacity are 21.273 and 15.652% respectively, and both have significant weight in rural public health emergency management capacity.

**Figure 2:**
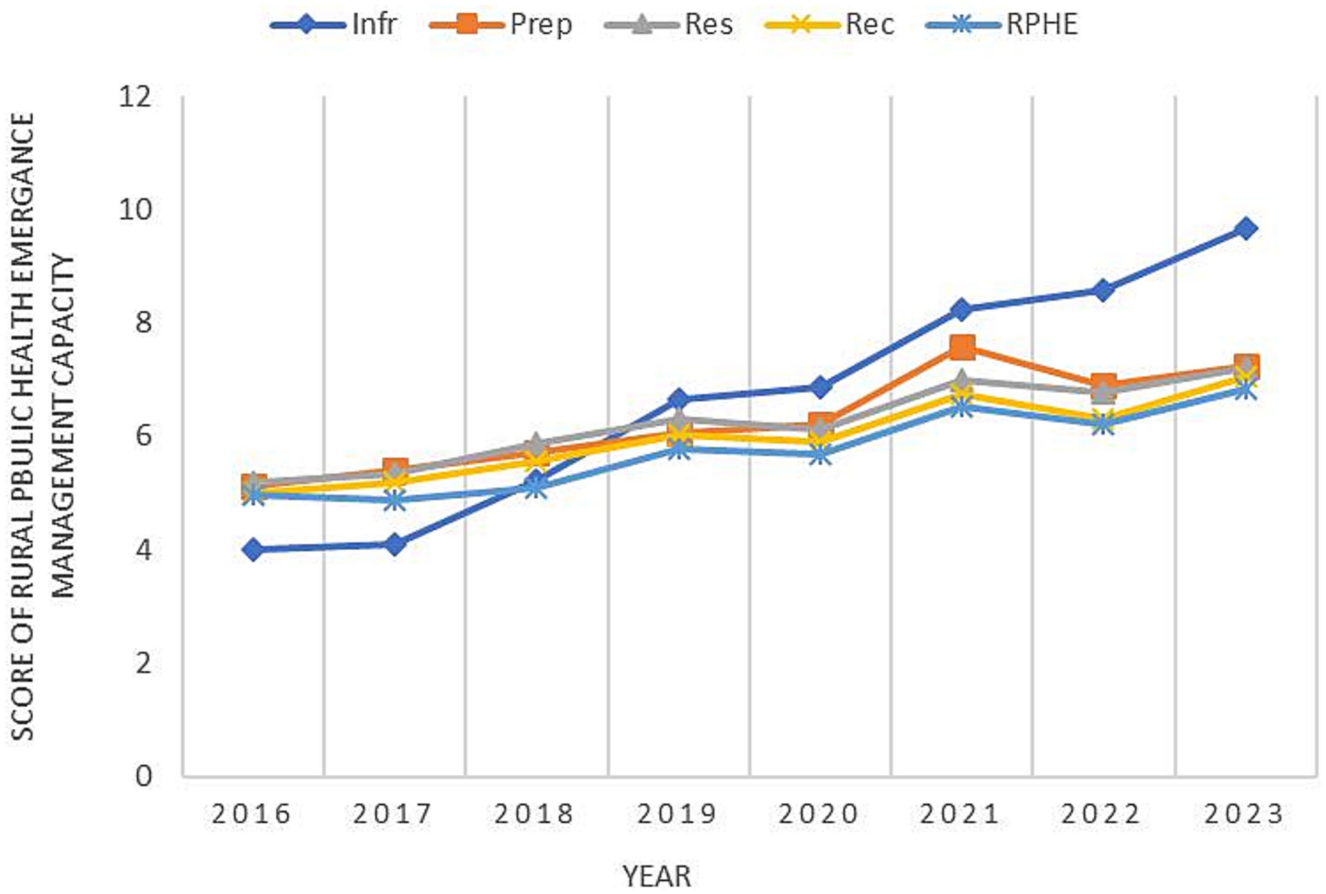
Changes in the score of rural public health emergency management capacity.

As shown in Figure 2, the graph of changes in the rural public health emergency management capacity index represents the line lines of changes in the emergency infrastructure capacity index, emergency preparedness capacity index, emergency response capacity index, emergency recovery capacity index and comprehensive index from 2016 to 2023, respectively. Overall, the composite index of rural public health emergency management capacity shows an upward trend year by year. The combined capacity score rose from 4.97 to 6.84. The growth of this index is not only an improvement in a single capacity, but also a manifestation of enhanced synergy among the four capabilities. From the perspective of stage changes, the period from 2016 to 2019 was the initial construction stage of the rural public health emergency management system. All capability indices fluctuated slightly between 5 and 6 zones, and the improvement of the four capabilities was relatively slow. The outbreak of the COVID-19 pandemic in 2020 became a key point. From 2020 to 2021, it was a period of rapid improvement, with all four capability indices showing significant enhancements. In 2022–2023, it entered a steady development stage. The growth rate slowed down compared with 2020–2021, but the resource capacity still maintained a relatively fast growth rate. From the perspective of differences in various dimensions, the growth rate of resource capability was the largest, rising from 4.02 points to 9.66 points. In 2016 and 2017, the score of resource capability was lower than the comprehensive index, but it began to be higher than the comprehensive index in 2018. The growth rate of resource capability slowed down from 2019 to 2020, reaching the highest value of 9.66 in 2023. Although the score of preparatory ability has always been higher than that of comprehensive ability, its growth rate was relatively slow from 2018 to 2020. It reached its peak in 2021, then declined in 2022, and rose to 7.23 again in 2023. The disposal capacity grew at a relatively fast rate from 2016 to 2019. In 2020, due to the impact of the COVID-19 pandemic, the disposal capacity dropped to 6.12, then rapidly rose to 6.99, briefly declined in 2022, and increased to 7.21 in 2023. The resilience index showed an upward trend from 2016 to 2019, slightly declined in 2020, rose rapidly in 2021, dropped again in 2022, and then rose to 7.05.

### 4.2 Discussion

This study attempts to conduct a comparative study on the emergency management capabilities of rural areas in response to public health events before and after the COVID-19 pandemic, dividing the rural public health emergency management capabilities into the pre-COVID-19 stage (2016–2019) and the post-COVID-19 stage (2020–2023) with 2020 as the time line. Through the changes in the four secondary indicators of emergency infrastructure capacity, emergency preparedness capacity, emergency response capacity and emergency recovery capacity and the combined score, and the compound annual growth rate of each capacity score, a systematic comparative analysis was conducted.

**Figure 3:**
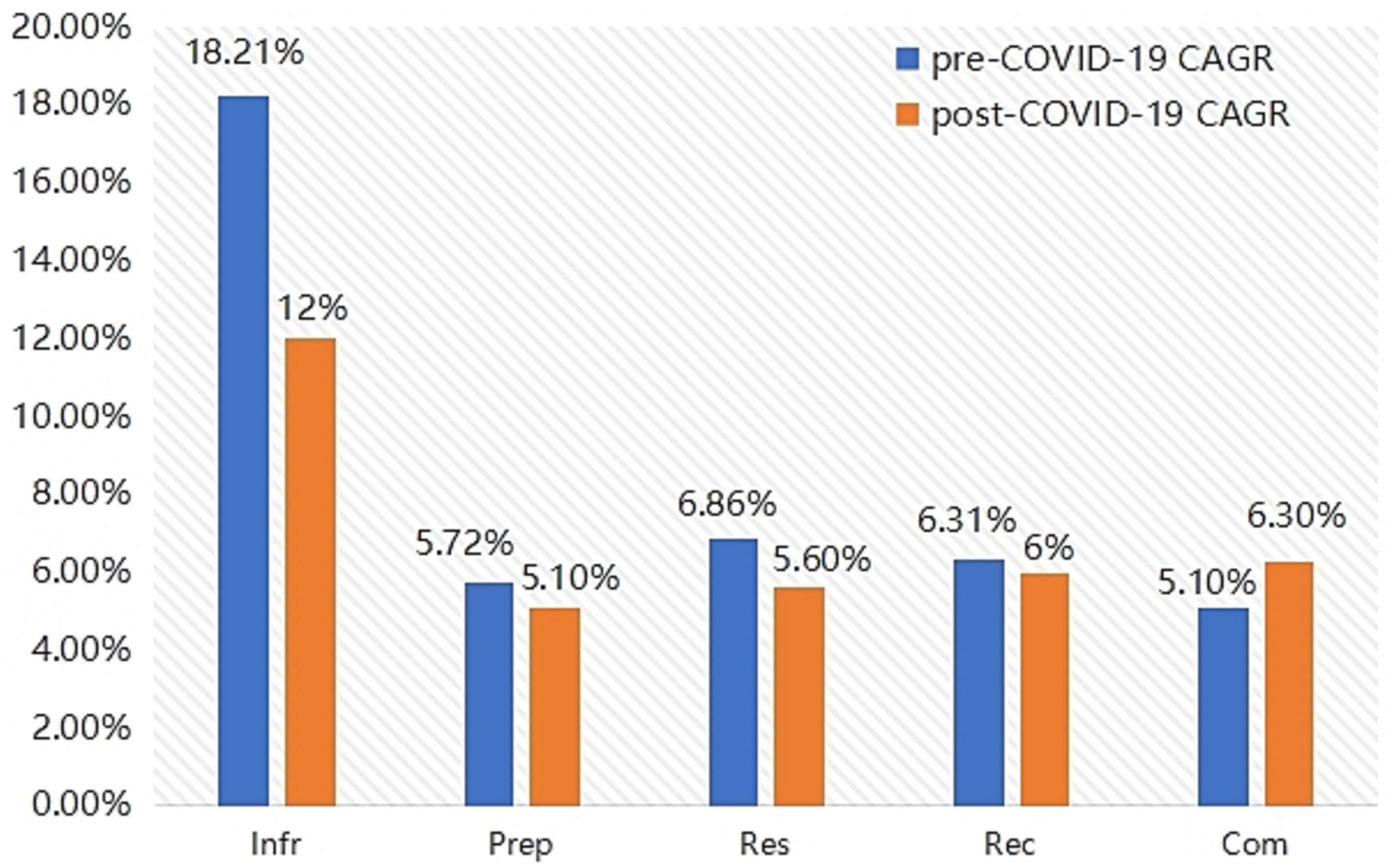
Compound annual growth rate of capabilities before and after COVID-19 (%).

As shown in Figure 3, the compound annual growth rate declined after COVID-19. Yet, absolute scores were consistently higher than in the pre-pandemic stage. This indicates that while expansion slowed, capacities became more consolidated and institutionalized, reflecting a shift from rapid deficiency-filling to quality-oriented governance.

The pandemic was accompanied by a systematic improvement in rural public health emergency response capabilities. From the analysis of compound annual growth rates, it can be seen that although the growth rates of the four capabilities declined slightly after the outbreak, the overall capacity level has significantly improved, especially the growth rate of the comprehensive capacity has accelerated after the outbreak. The compound annual growth rate of the combined capabilities rose from 5.1 to 6.4%, showing a clear growth trend. This indicates that in rural areas, the level of public health emergency management has improved after the COVID-19 pandemic. The observed patterns are consistent with the emergence of a more stable and sustainable emergency management mechanism appears to have emerged, which is consistent with a possible transformation from passive response to active governance.

Structural explanations behind the slowdown in average annual growth rate. The average annual growth rate of emergency infrastructure, emergency preparedness, emergency response and emergency recovery capacity has slowed since the outbreak compared with that before the outbreak. This does not imply a decline in capacity, but reflects the evolution of capacity from making up for deficiencies at a high speed to improving steadily at a high quality. Table 8 compares structural changes before and after COVID-19. It highlights that infrastructure grew rapidly from a low base, while preparedness and recovery shifted toward steady, quality-oriented growth. This structural divergence suggests that post-pandemic improvements were not uniform but differentiated across capacity types.

(1) Emergency infrastructure capacity. Before the pandemic, the average annual growth rate of emergency infrastructure capacity was as high as 17.4%, but the base was relatively low in the early stage, reflecting a serious shortage of public health resources at the grassroots level, with obvious shortcomings in medical infrastructure, staffing, etc. After the pandemic, the compound annual growth rate dropped to 12.0%, indicating that emergency infrastructure capacity has gradually entered a plateau period, showing a structural shift from high-speed growth to stable accumulation. But the absolute growth was significant, especially when the emergency infrastructure capacity index peaked at 9.66 in 2023, reflecting that during the pandemic, the country shifted a large amount of medical supplies, public health infrastructure and health human resources to rural areas. The rapid increase in emergency infrastructure capacity provided the material basis for subsequent emergency response and recovery (25).
(2) Emergency preparedness capacity. The compound annual growth rate of the emergency preparedness capacity index dropped from 5.6% before the pandemic to 3.8% after the pandemic, indicating that in the stage of regular governance, emergency preparedness capacity building pays more attention to quality and norms, and the policy focus shifts from quantity to quality, which will stabilize the compound annual growth rate. However, the capacity index has developed to 7.23, indicating that the capacity building has entered a relatively high level platform. The outbreak has prompted the government to quickly build up contingency plans, training mechanisms, and material allocation systems, and to move emergency preparedness capacity from emergency response to regular governance (26). In reality, the outbreak has endangered people’s lives and safety, forcing localities to establish more scientific and systematic emergency response plans. This enhanced capacity indicates that rural areas have gradually acquired strategic preparedness to deal with major public health events.
(3) Emergency response capacity. The compound annual growth rate of rural emergency response capacity was 5.3% before the outbreak, but dropped to 4.4% after the outbreak. This indicates that the focus of capacity building in the later stage of the epidemic was institutional optimization and process refinement, with relatively reduced marginal improvement space and naturally slower pace. However, the rise of the capacity index to 7.21 in 2023 indicates that rural areas have improved their ability to accurately judge the situation, report in a timely manner, and communicate effectively during the pandemic. Under the leadership of grassroots Party organizations, the collaborative response mechanism among grassroots governments, village doctors, grid workers and other groups has been exercised. Rural public health emergency response has shifted from “slow action” to “quick response and clear division of labor.”
(4) Emergency recovery capacity. The average growth rate of emergency recovery capacity after the outbreak of COVID-19 was lower than that before the outbreak, but the growth rate changed slowly, indicating that after the outbreak, the government expanded the coverage of the social security system and improved the mechanism for the continuation of medical services, the resilience of rural areas in terms of post-disaster psychological intervention and economic recovery was enhanced. At the same time, the government has been coordinating poverty alleviation with rural revitalization, promoting a steady increase in rural residents’ income and improving the post-disaster recovery system. However, in rural areas, due to weak basic resources, high population mobility, poor information flow and other shortcomings, there is much reliance on policy guidance from higher authorities during post-disaster recovery. It is suggested that in the future, efforts should be focused on building long-term mechanisms for emergency recovery in rural areas, such as rural psychological support networks, collaborative mechanisms for resumption of work and production, and post-epidemic information disclosure platforms.

**Table 8:** Comparison of the evolution of capacity structure for rural public health emergency management before and after the COVID-19 Pandemic.

Dimensions	Pre-pandemic development characteristics (2016–2019)	Post-pandemic development characteristics (2020–2023)	Transformation trends
Infr	High growth, low base	The growth rate slowed, but the absolute value increased significantly	From filling the gaps to strengthening the foundation
Prep	Lack of system and rough contingency plans	Regular governance is gradually being established	From emergency to regular governance
Res	Slow response and inadequate coordination mechanism	Synergy and improvement, the system is gradually becoming more complete	From dispersion to system governance
Rec	Post-disaster recovery relies on superior resources	Social security has been expanded and its resilience has been enhanced	From dependence to autonomy

In addition to describing numerical trends, this study also emphasizes the underlying social and governance logic. The COVID-19 pandemic not only accelerated the quantitative improvement of rural emergency indicators but also promoted the rise of public health awareness, the popularization of digital technologies, and the gradual institutionalization of governance practices. These changes resonate with the concepts of “collaborative governance” and “resilience theory,” suggesting that the enhancement of rural public health emergency capabilities represents not merely numerical growth but also a systematic evolution of governance models.

## 5 Conclusions and recommendations

### 5.1 Conclusion

This paper selects four indicators—emergency infrastructure capacity, emergency preparedness capacity, emergency response capacity, and emergency recovery capacity—to measure the emergency management capacity of rural public health, conducts a comparative analysis of the changing trends of capacity before and after the COVID-19 pandemic, and draws the following conclusions:

The COVID-19 pandemic coincided with significant improvements in rural public health emergency infrastructure capacity, which may have acted as an external catalyst: Since the outbreak of COVID-19 in 2020, the compound annual growth rate of comprehensive capacity has gradually increased from 5.1 to 6.4%, reflecting the continuous improvement of the comprehensive capacity of the rural emergency response system in dealing with public health emergencies and the continuous improvement of the efficiency of emergency response. Rural public health emergency management has undergone a qualitative change from passive response to active evolution in terms of institutional building and governance logic, a finding that theoretically confirms the logic of resilience governance, namely that under major shocks, institutions, culture, and social structures jointly drive the evolution of governance capacity.

The improvements observed after the pandemic are consistent with a shift from fragmented governance to systematic governance: Before the outbreak of COVID-19, rural public health emergency management in Jiangsu Province was characterized by fragmented and partial development, mainly relying on administrative means and phased policy support to enhance rural public health emergency management capabilities, and the rural public health emergency system lacked continuous construction and systematic planning. After the outbreak, a large amount of resources such as policies, finance, infrastructure, etc. were rapidly transferred to rural areas, and emergency management was gradually embedded in the rural governance system, forming a capacity structure that integrates “resources, preparedness, disposal, and recovery.” This process indicates that rural public health governance is shifting away from traditional administrative and fragmented patterns toward a more systematic and collaborative model.

The epidemic has promoted the transformation of rural public health emergency governance toward “regularized—law-based—coordinated”: although the compound annual growth rate has slowed overall after the epidemic, it reflects the deep evolution of the rural public health emergency system. After the outbreak of COVID-19, most towns in rural areas of Jiangsu Province have established and improved regular drill mechanisms, information reporting mechanisms and designated medical resource allocation plans, and the emergency response system has shifted from post-event response to pre-event preparation. The pandemic has to some extent promoted the implementation of regulations such as the “Regulations on Emergency Response to Public Health Emergencies” at the grassroots level, and the public health emergency response system has developed in an institutionalized direction. The pandemic has given rise to a mechanism of cooperation among multiple governance entities. Local governments have joined forces with village committees, village doctors, volunteers and social organizations to respond to the COVID-19 pandemic, demonstrating a coordinated response strategy that reflects not only the improvement of institutional arrangements but also the enhancement of socio-cultural awareness and grassroots governance logic.

### 5.2 Recommendations

Based on the analysis of the evaluation index system for the capacity of public health emergency management in villages in our country, we put forward the following three suggestions:

(1) Increase financial support to strengthen the supply of rural medical resources. First of all, relevant departments should attach importance to the construction of rural public health institutions, improve the infrastructure construction level for treating major infectious diseases, and increase investment in medical equipment, wards and beds. We should do a good job in building up the reserve of medical and health personnel, especially the system guarantee system for medical and health personnel at the grassroots level in rural areas. Secondly, regarding the allocation of funds, it should be made clear that government investment is the main channel of investment for public health institutions, and financial input for public health institutions should be implemented, especially for county-level public health institutions, more investment should be made to make public health institutions truly full budget-funded units and ensure the effective functioning of public health (27). At the implementation level, it is recommended that county-level finance and health departments take the lead in overall planning, while township health centers and village committees are responsible for execution, so as to ensure the precise allocation of financial and human resources.
(2) Improve the emergency response plans at the grassroots level and make adequate preparations. The work of rural public health emergency response plans is the prerequisite for effectively responding to public health emergencies. First of all, the grassroots public health emergency response plan should be improved and updated dynamically based on the actual situation to ensure that the content of the plan is scientific and reasonable, comprehensive in coverage and operational. Secondly, regularly organizing emergency drills and training is the key to preparedness. In light of practical needs, on-site simulation exercises and cross-departmental joint exercises should be carried out to enhance the emergency response capabilities of grassroots health workers. In practice, it is advisable to stipulate at least two cross-departmental joint drills per year and establish measurable assessment indicators to evaluate the effectiveness of emergency plans.
(3) Build multi-structural collaborative cooperation to enhance emergency response capabilities. First, relevant departments still need to improve the operation mechanism of rural public health emergency management, establish the concept of collaborative governance, further promote the decentralization of public power, and enhance the consistency of rights and responsibilities in emergency resource management at the grassroots level. Secondly, local governments should break away from traditional ideas. Public health work should follow the emergency work policy of prevention first, prevention and control coordination, peacetime and wartime integration, and constant preparedness, and adhere to the principle of unified leadership of the Party committee, hierarchical responsibility and guidance of relevant departments, and joint participation of social organizations. Finally, it is necessary to clarify the boundaries of governance rights and responsibilities, so that the government can play a leading role in public health emergency management and coordinate the efforts of all parties. Social organizations should play to their strengths, assume relevant responsibilities, and provide supportive services, while grassroots communities and residents actively participate in public health protection. To ensure that collaborative governance is effectively implemented, it is recommended to establish inter-departmental joint conference systems and normalized communication mechanisms.
(4) Improve the mechanism for handling public health incidents and enhance residents’ sense of happiness. First of all, the government should focus on fairness and maintain public trust through the use of evidence-based interventions and completely transparent, fact-based communication (28). Introduce policies to support the recovery and reconstruction of rural social organizations, such as reducing rent for their premises, providing interest-free or low-interest loans, etc., to relieve financial pressure and restore normal operations. Social organizations can actively cooperate with social enterprises and obtain financial support from social enterprises by providing services to them (29, 30). Secondly, promote the informatization of rural public health, promote the transmission and sharing of information after emergency response through new technologies such as the Internet and big data, and establish a comprehensive information system and a flat information management mechanism. Finally, the government should revitalize rural areas, retain all kinds of talents and labor force, and avoid the shortage of human resources in rural social organizations during the recovery stage by improving the internal salary system. At the same time, a third-party evaluation mechanism should be introduced to monitor and provide feedback on policy implementation, enabling timely adjustments and optimization. Additional Requirements.

This study inevitably has some limitations. First, it exclusively focuses on Jiangsu Province, a relatively developed region in China. Therefore, the conclusions cannot be directly generalized to other provinces, especially those with different resource endowments, governance capacity, and infrastructure conditions. Future studies should conduct inter-provincial comparisons between eastern, central, and western provinces to test whether the observed patterns hold in different contexts. Second, the evaluation relies heavily on expert scoring, which may introduce subjectivity despite the strict control measures taken; future studies could adopt triangulation approaches such as combining questionnaire surveys, field interviews, and secondary data to enhance robustness. Third, the study is mainly based on quantitative analysis, lacking in-depth qualitative insights. Future research could incorporate interviews with grassroots cadres, village doctors, and residents to reveal the deeper institutional dynamics and social cognition. Finally, although the study connects its findings with governance and resilience theory, further theoretical integration with concepts such as multi-level governance or institutionalism may provide richer explanatory power.

## Data Availability

The original contributions presented in the study are included in the article/supplementary material, further inquiries can be directed to the corresponding author.
